# Reoperations for structurally persistent or recurrent disease after thyroidectomy: analysis via preoperative CT

**DOI:** 10.1038/s41598-020-69398-w

**Published:** 2020-07-23

**Authors:** Hyeung Kyoo Kim, Eun Ju Ha, Miran Han, Jeonghun Lee, Euy Young Soh

**Affiliations:** 1 Department of Surgery, Ajou University School of Medicine, Wonchon-Dong, Yeongtong-Gu, Suwon, 443-380 Korea; 2 Department of Radiology, Ajou University School of Medicine, Wonchon-Dong, Yeongtong-Gu, Suwon, 443-380 Korea

**Keywords:** Diseases, Endocrinology

## Abstract

The incidence rates of structural persistent disease (PD) and recurrent disease (RD) after thyroidectomy, and their clinicoradiological (CT) characteristics, remain poorly understood. Therefore, we characterized differentiated thyroid cancer (DTC) patients who underwent re-operations, with a focus on preoperative CT scans. We examined neck CT scans obtained prior to initial surgery and reoperation, and classified the disease into four categories according to the persistence/recurrence and neck dissection/non-dissection status. In total, 121 of 9,173 DTC patients underwent reoperations to treat PD or RD; the mean time to reoperation was 25.5 and 54.1 months, respectively. Of all reoperations, 19% (23/121) were performed to treat RD; 81% (98/121) were performed to treat PD. Compared to RD, PD was commonly detected in the non-dissected neck. Tumor multiplicity and the number of pathologically positive lymph nodes were greater in the non-dissected than dissected neck. A review of the CT data revealed more false-negative findings on the 60-s- versus 30–40-s-delay scans of PD patients with non-dissected necks. In conclusion, most of the reoperations performed on DTC patients were for management of PD. Improved preoperative CT assessments and initial surgery, based on the information of clinico-radiological characteristics, are required in the care of DTC patients.

## Introduction

The incidence of thyroid cancer has increased substantially in recent decades^1^. Accurate preoperative staging and surgical planning are essential to optimize outcomes and prevent locoregional recurrence^2,3^. Although differentiated thyroid cancers (DTCs) are associated with good prognoses and a low mortality rate, cervical lymph node metastases have been reported in 60–70% of patients in association with locoregional recurrence^4–6^. Therefore, the current guidelines recommend preoperative computed tomography (CT) with intravenous contrast for patients clinically suspected of advanced disease^2,3^. This imaging modality is used to evaluate the preoperative status and facilitates complete removal of cancers.

However, structurally persistent disease (PD) and recurrent disease (RD) remain challenging and have a combined incidence of up to 30% after thyroidectomy^7,8^. Age, gender, tumor size, histological findings, and cervical lymph node metastasis are all associated with the risk of recurrence^2,9–11^. The American Thyroid Association (ATA) guidelines state that mutational status and vascular invasion of follicular thyroid cancer are also prognostic factors^2^. However, although DTCs are generally stable or slow-growing, recurrence often develop rapidly after surgery, such that PD is problematic in some cases^8,12^. Currently, the incidence rates of structural PD and RD, and their clinicoradiological (CT) characteristics, remain poorly understood. Therefore, we characterized DTC patients who underwent re-operations in terms of the incidence rates and clinicoradiological characteristics of PD and RD, with a focus on preoperative CT scans. We retrospectively reviewed 17 years of medical records held in a single tertiary hospital.

## Results

### Patient demographics and characteristics after initial surgery

Table 1 summarizes the baseline clinicopathological characteristics of the patients with structural PD and RD after the initial surgery. The mean age at diagnosis was 44.5 years (range: 14–78 years) and the mean initial tumor size was 1.7 ± 1.3 cm (range: 0.2–9.0 cm). The mean follow-up duration after surgery was 76 ± 38 months. Almost all of the patients underwent surgery to treat conventional papillary thyroid cancer (117/121, 96.7%). Compared to patients with PD, those with RD were more often likely to be male (*P* = 0.026) and to have unilateral disease (*P* = 0.042). We found no significant difference in age, tumor size or histology, the extent of gross extrathyroidal extension or lymphovascular invasion, TNM stage, or the extent of initial surgery between patients with PD and RD.

**Table 1:** Clinicopathological characteristics of all patients.

Characteristic	Persistent disease (n = 98)	Recurrent disease (n = 23)	Total (n = 121)	*P*-value
Mean age at diagnosis (years)	44.7 ± 13.5	43.6 ± 13.8	44.5 ± 13.5	0.721
< 55	75 (76.5)	19 (82.6)	94 (77.7)	
≥ 55	23 (23.5)	4 (17.4)	27 (22.3)	
Gender (male: female)	24:74	11:12	35:86	0.026
Initial tumor size (cm)	1.7 ± 1.2	1.8 ± 1.9	1.7 ± 1.3	0.765
< 1.0	24 (24.5)	9 (39.1)	33 (27.3)	
≥ 1.0	74 (75.5)	14 (60.9)	88 (72.7)	
Histological findings				1.000
Conventional PTC	94 (95.9)	23 (100)	117 (96.7)	
Follicular variant	1 (1.0)	0 (0)	1 (0.8)	
Tall cell variant	1 (1.0)	0 (0)	1 (0.8)	
Diffuse sclerosing variant	2 (2.0)	0 (0)	2 (1.7)	
Bilaterality	44 (44.9)	5 (21.7)	49 (40.5)	0.042
Gross extrathyroidal extension	14 (14.3)	1 (4.3)	15 (12.4)	0.298
Lymphovascular invasion	25 (25.5)	3 (13.0)	28 (23.0)	0.276
Initial TNM stage (8^th^ edition)				0.567
I	77 (78.6)	19 (82.6)	96 (79.3)	
II	19 (19.4)	4 (17.4)	23 (19.0)	
III	2 (2.0)	0 (0)	2 (1.7)	
IV	0 (0)	0 (0)	0 (0.0)	
Extent of initial operation				0.132
Lobectomy with CND	10 (10.2)	1 (4.3)	11 (9.1)	
Total thyroidectomy with CND	45 (45.9)	8 (34.8)	53 (43.8)	
Total thyroidectomy with ipsilateral MRND	29 (29.6)	9 (39.1)	38 (31.4)	
Total thyroidectomy with bilateral MRND	14 (14.3)	5 (21.7)	19 (15.7)	

Values are shown as mean ± standard deviation. The numbers in parentheses are percentages.

*PTC* papillary thyroid carcinoma, *CND* central neck dissection, *MRND* modified radical neck dissection, *TNM* tumor, node, and metastases.

### Relative incidences and clinicoradiological characteristics of PD and RD

Table 2 shows the incidence rates and clinicoradiological characteristics of patients with structural PD and RD. Of the 121 patients, PD was evident in 81.0% (98/121) and RD in 19% (23/121). The mean time to reoperation was 25.5 months for PD patients and 54.1 months for RD patients (*P* < 0.001). PD was found in the previously dissected neck in 32.7% of patients (32/98), and in the non-dissected neck in 67.3% of patients (66/98). RD was evident in the previously dissected neck in 56.5% of patients (13/23), and in the non-dissected neck in 43.5% of patients (10/23). Compared to RD, PD was more commonly detected in the non-dissected neck (*P* = 0.033). In both groups, tumor multiplicity and the number of pathologically positive lymph nodes after reoperation were significantly greater in the non-dissected than dissected neck (both, *P* < 0.05).

**Table 2:** Clinical and radiological characteristics of patients with persistent disease and recurrent disease.

Characteristic	Persistent disease				Recurrent disease				*P*-value
	Total (n = 98)	Neck dissected (n = 32)	Neck not dissected (n = 66)	*P*-value	Total (n = 23)	Neck dissected (n = 13)	Neck not dissected (n = 10)	*P*-value	
Time to reoperation (months)	25.5 ± 18.4	21.1 ± 18.7	27.7 ± 18.0	0.093	54.1 ± 24.1	58.9 ± 29.0	48.0 ± 15.0	0.260	< 0.001
Tumor multiplicity	62 (63.3)	14 (43.8)	48 (72.7)	0.005	16 (69.6)	7 (53.8)	9 (90.0)	0.089	0.570
Location of disease				0.013				0.046	0.019
Central compartment	6 (6.1)	5 (15.6)	1 (1.5)		5 (21.7)	5 (38.5)	0 (0.0)		
Ipsilateral compartment	79 (80.6)	20 (62.5)	59 (89.4)		17 (73.9)	7 (53.8)	10 (100.0)		
Bilateral compartment	13 (13.3)	7 (21.9)	6 (9.1)		1 (4.3)	1 (7.7)	0 (0.0)		
Number of pathologically positive LNs	3.0 ± 2.8	2.1 ± 2.0	3.5 ± 3.0	0.017	2.5 ± 1.6	1.9 ± 1.0	3.4 ± 1.8	0.027	0.388

Values are shown as mean ± standard deviation. The numbers in parentheses are percentages.

*LN* lymph node.

### PD and RD in the dissected neck

Among patients with a dissected neck, the mean time to reoperation was significantly higher in those with RD than PD (21.1 vs. 58.9 months; *P* < 0.001). However, no other clinical or radiological feature significantly predicted PD. A retrospective review of the CT scans showed that both RD and PD were most frequently located between the common carotid artery and the internal jugular vein, followed by the low-level VI–VII, high-level II, and level V regions, the retropharyngeal space, and the supraclavicular fossa (Table 3).

**Table 3:** Clinical and radiological characteristics of patients with persistent disease and recurrent disease who underwent neck dissection, based on computed tomography.

Characteristic	Total (n = 45)	Persistent disease (n = 32)	Recurrent disease (n = 13)	*P*-value
Time to reoperation (months)	32.0 ± 27.8	21.1 ± 18.7	58.9 ± 29.0	< 0.001
Tumor multiplicity	21 (46.7)	14 (43.8)	7 (53.8)	0.538
Location				0.095
Central compartment	10 (22.2)	5 (15.6)	5 (38.5)	
Ipsilateral compartment	27 (60.0)	20 (62.5)	7 (53.8)	
Bilateral compartment	8 (17.8)	7 (21.9)	1 (7.7)	
Number of pathologically positive LNs	2.0 ± 1.7	2.1 ± 2.0	1.9 ± 1.0	0.668
**Tumor location**				
Between the CCA and IJV	18 (40.0)	13 (40.6)	5 (38.5)	
Supraclavicular area	2 (4.4)	1 (3.1)	1 (7.7)	
High-level II region	7 (15.6)	5 (15.6)	2 (15.4)	
Low-level VI/VII regions	9 (20.0)	5 (15.6)	4 (30.8)	
Level V	5 (11.1)	4 (12.5)	1 (7.7)	
Retropharyngeal space	4 (8.9)	4 (12.5)	0 (0.0)	

Values are shown as mean ± standard deviation. The numbers in parentheses are percentages.

*CCA* common carotid artery, *IJV* internal jugular vein, *LN* lymph node.

### PD and RD in non-dissected necks

**Figure 1:**
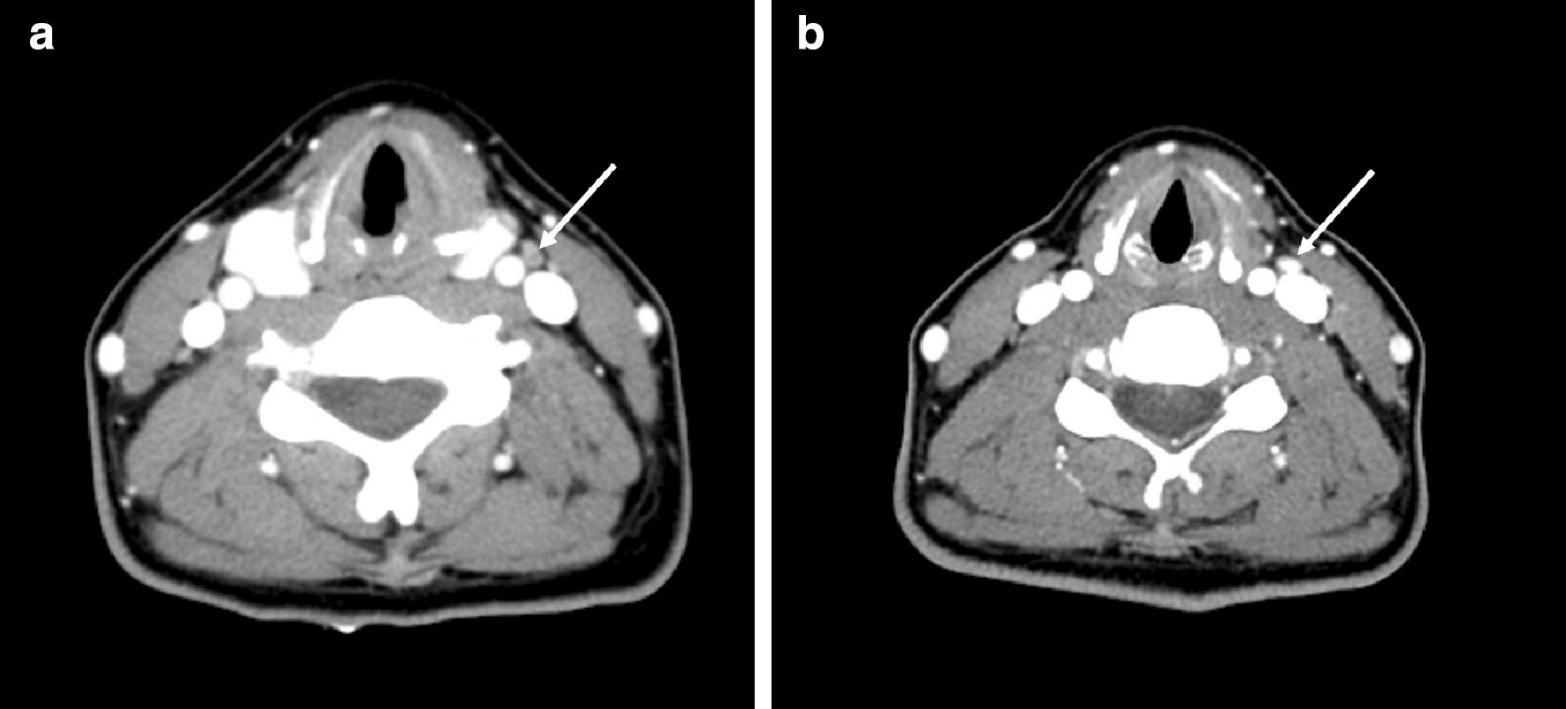
Persistent disease in a 43-year-old woman with bilateral thyroid cancers. (**A**) Axial CT image with a 60-s scan delay shows a small lymph node at left level III (arrow) without suspicious CT features before initial surgery. She underwent total thyroidectomy with central neck dissection. (**B**) Axial CT image with a 40-s scan delay shows a lymph node (arrow) with a strong enhancement at the same location before reoperation.

In patients with a non-dissected neck, the mean time to reoperation was significantly longer in those with RD than PD (27.7 vs. 48.0 months; *P* = 0.001). A retrospective review of the CT scans showed that 42.4% of the PD patients (28/66) had false-negative findings. Among the 66 PD patients, false-negative findings were more frequent on the 60-s-delay scans (48.1%, 25/52) than the 30–40-s-delay scans (21.4%, 3/14) (Table 4) (Fig. 1). Among the suspicious lymph node findings, abnormal enhancement pattern (focally strong or heterogeneous enhancement) was missed most frequently, followed by cystic change, and calcification (Table 5).

**Table 4:** Clinical and radiological characteristics of patients with persistent and recurrent disease in the non-dissected neck based on computed tomography.

Characteristic	Total (n = 76)	Persistent disease (n = 66)	Recurrent disease (n = 10)	*P*-value
Time to reoperation (months)	30.4 ± 18.8	27.7 ± 18.0	48.0 ± 15.0	0.001
Tumor multiplicity	57 (75.0)	48 (72.7)	9 (90.0)	0.436
Location				1.000
Central compartment	1 (1.3)	1 (1.5)	0 (0.0)	
Ipsilateral compartment	69 (90.8)	59 (89.4)	10 (100.0)	
Bilateral compartment	6 (7.9)	6 (9.1)	0 (0.0)	
Number of pathologically positive LNs	3.5 ± 2.8	3.5 ± 3.0	3.4 ± 1.8	0.918
**CT delays**				
Protocol A (60-s-delay scan)	59 (77.6)	52 (78.8)	7 (70.0)	0.012
Positive findings	27 (45.8)	27 (51.9)	0 (0.0)	
Negative finding	32 (54.2)	25 (48.1)	7 (100.0)	
Protocol B (30/40-s-delay scan)	17 (22.4)	14 (21.9)	3 (30.0)	0.029
Positive findings	11 (64.7)	11 (78.6)	0 (0.0)	
Negative findings	6 (35.3)	3 (21.4)	3 (100.0)	

Values are shown as mean ± standard deviation. The numbers in parentheses are percentages.

*LN* lymph node.

**Table 5:** Retrospective computed tomography imaging features of persistent disease patients who did not undergo neck dissection.

Variable	Total (n = 66)	Protocol A (n = 52)	Protocol B (n = 14)
False-negative findings on CT	28 (42.4)	25 (48.1)	3 (21.4)
CT-positivity missed	38 (57.6)	27 (51.9)	11 (78.6)
Calcification	2 (5.3)	2 (7.4)	0 (0.0)
Cystic/necrotic change	8 (21.1)	6 (22.2)	2 (18.2)
Focally strong or heterogeneous enhancement	28 (73.7)	19 (70.4)	9 (81.8)

The numbers in parentheses are percentages. Protocol A refers to a 60-s scan delay after intravenous injection (IV) of 100 mL iodinated contrast agent; and Protocol B refers to a 30- or 40-s scan delay after IV injection of 90 mL iodinated contrast agent.

*CT* computed tomography.

### Treatment responses after reoperation

Regarding the clinical outcomes after reoperation, we excluded 12 patients with interfering antibodies; we evaluated the treatment responses of 109 patients after re-operation; 83.5% (91/109) exhibited excellent responses. We found no significant difference between PD and RD patients.

## Discussion

Among our patients, the incidence of RD was only 19% (23/121), while that of PD was 81.0% (98/121), as revealed by CT. Of the 121 patients, 52.1% (63) required reoperation within the first 2 years, reflecting the high incidence of PD, most commonly in the non-dissected neck (67.3%, 66/98). Improved preoperative CT assessments and initial surgery, based on the information of clinico-radiological characteristics, are required in the care of DTC patients.

Although DTC has an excellent survival rate, recurrence remains a major concern; up to 30% of patients develop recurrence^7,8^. Differential diagnosis of RD or PD can be difficult. Currently, all reoperations performed after initial therapy are considered to reflect recurrence. However, as the average time to recurrence has been reported to range from 6 months to several decades^4,13,14^, several studies have accepted that PD is also a factor in recurrence^8,12^. The following factors are associated with the risk of recurrence (without structural disease) after initial surgery: lymph node metastasis, histological findings, tumor size, extrathyroidal and extranodal extensions, sex, and age at diagnosis^2,9–11^. Based on these factors, the revised ATA guidelines suggest that the risk of DTC should be stratified as low, intermediate, and high^2^. However, the incidence rates and time of onset of RD and PD remain unclear. Preoperative CT yields detailed information on nodal location by reference to anatomical surgical landmarks. Therefore, we used neck CT scans acquired before the initial surgery and reoperation to determine whether reoperated patients exhibited PD or RD; almost all DTC patients undergoing reoperation exhibited PD. The mean time to reoperation was significantly different between RD patients (54.1 months) and PD patients (25.5 months). Thus, we consider that the cause of PD must be investigated when clinically examining a patient requiring reoperation within 2 years after initial operation.

It is clear that PD developing after DTC surgery reflects inadequate initial surgery. Several studies have identified parameters contributing to the risk of disease persistence^8,12^. We found that PD developed in 67.3% of patients who did not undergo neck dissection, strongly suggesting that improvements in preoperative CT assessment and initial surgery are essential. In terms of preoperative assessment via CT, several studies have shown that scan delays affect tumor conspicuity^15,16^. Lee et al. reported that thyroid tumors were more conspicuous on early 40-s-delay (rather than 70-s-delay) scans^15^. Park et al. reported that 25-s-delay CT scans improved the diagnostic accuracy of thyroid cancer-associated lateral lymph node metastases^16^. We found that false-negative CT findings in PD patients were more common when 60-s-delay rather than 30–40-s-delay scans were examined, emphasizing that early CT scans are optimal for identifying malignant lymph nodes exhibiting focal strong or heterogeneous enhancement. However, in clinical practice, there are cases in which the routine neck CT scan (60-s-delay scan) is taken in preoperative assessment of thyroid cancer patients. Contrast-enhanced CT protocols should be optimized for improving the diagnostic accuracy for cervical lymph node metastasis from thyroid cancer and the early phase (30–40 s-delay) scans are necessary for minimizing the PD. However, 32.7% of the PD patients had undergone neck dissection, suggesting that tumors in certain locations had been incompletely removed. We consider that a multidisciplinary team including both radiologists and surgeons must review preoperative CT findings thoroughly and determine the extent of surgery required to improve surgical outcomes.

This study had some limitations. First, it used a retrospective design and we only had access to medical records data; this was particularly problematic with respect to establishing the cause of PD. Second, it may not be possible to distinguish growth of microscopic tumors not detected on initial imaging from true recurrence. Third, since we reclassified the TNM stage based on the revised AJCC 8^th^ edition, there may be some errors in determining the gross extrathyroidal extension. However, they were decided after thorough evaluation of the surgical and pathologic reports. Fourth, patients who went through RAI treatment after initial thyroidectomy could make an interference factor. However, since RAI treatment was performed in most patients after total thyroidectomy (105/110, 95.5%), the impact on the results is considered limited. Fifth, different surgeons and junior/senior surgeons may make a big difference in surgical technique, and this will make a great difference in clearance of neck dissections. Further research using a multicenter study design is required in the future.

In conclusion, in most cases herein, reoperation was performed as part of the management of PD revealed by CT. Improved preoperative CT assessments and initial surgery, using a multi-disciplinary team approach, are required in the care of DTC patients.

## Methods

### Study design and patients

**Figure 2:**
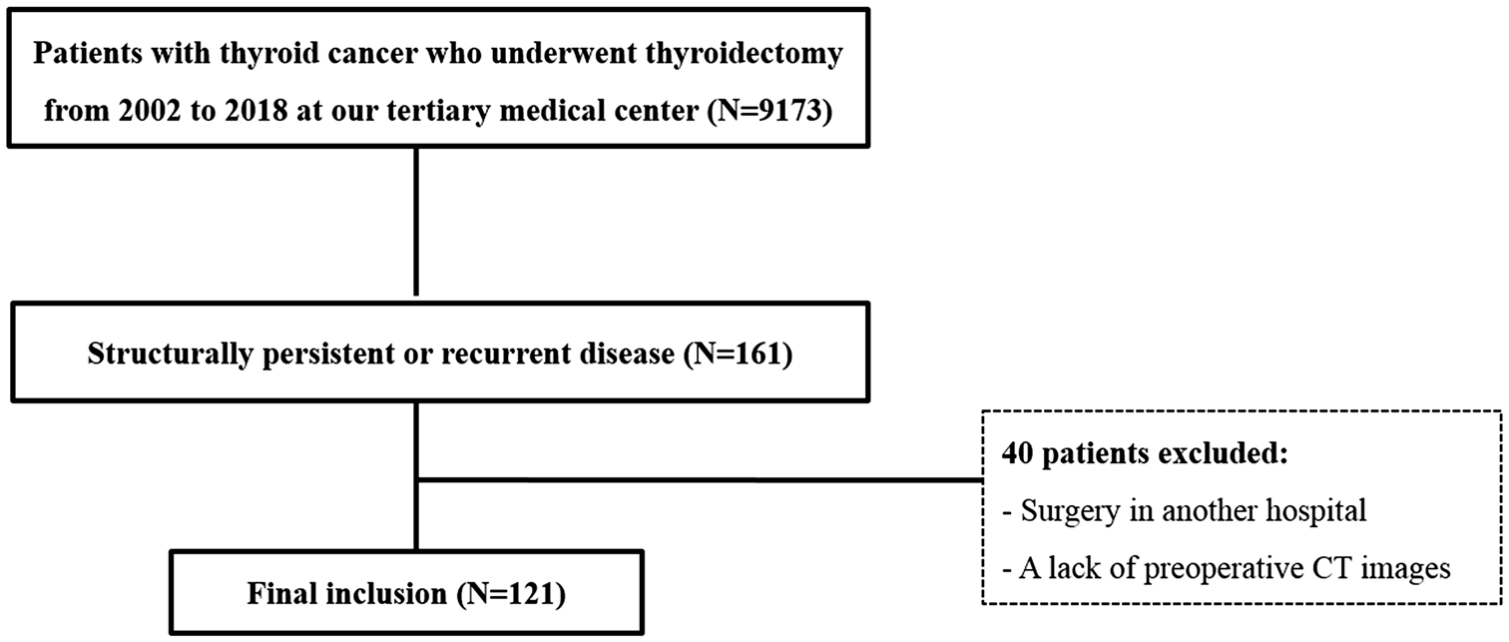
The flowchart of patient enrolment.

This study was approved by the Ajou University hospital Institutional Review Board (AJIRB-MED-MDB-19-424) and implemented in accordance with the ethical standards of the 1964 Declaration of Helsinki and its later amendments. Informed consent was obtained from all patients prior to neck CT, ultrasound (US)-guided biopsy, and operation. We retrospectively included 9,173 patients with thyroid cancer who underwent thyroidectomy from 2002 to 2018 at our tertiary medical center. Our database identified 121 DTC patients exhibiting structural PD or RD after initial surgery. The inclusion criteria were as follows: (1) cervical lymph node metastases from DTC, as confirmed by US-guided aspiration/biopsy, (2) subsequent neck dissection and (3) availability of final histopathological results. The exclusion criteria were (1) a lack of preoperative CT images and (2) surgery in another hospital. The flowchart of patient enrolment is shown in Fig. 2.

### CT protocol

Preoperative CT images were obtained using 64- or 128-channel multi-detector scanners (SOMATOM Definition Flash; Siemens Medical Solutions, Cary, NC, USA; Brilliance; Philips Medical Systems, Best, The Netherlands). The CT scans started at the aorticopulmonary window and continued toward the skull base. The CT images were obtained at 0.5–0.6 mm collimation and were reconstructed into axial images every 2.0 mm on a 512 × 512 matrix. During the study period, two CT protocols that differed in terms of contrast injection and image acquisition timing were used, based on a literature review and according to consensus among radiologists, as follows: (1) a 60-s scan delay after intravenous (IV) injection of 100 mL iodinated contrast agent (January 2002 to February 2013); and, (2) a 30- or 40-s scan delay after IV injection of 90 mL iodinated contrast agent (March 2013 to December 2018). For all scans, the same iodinated contrast agent (Omnihexol 300 [iohexol]; WiTH Healthcare, Korea) was injected into the right arm, followed by a flushing injection of 20–30 mL normal saline delivered at 3 mL/s using an automated injector.

### CT evaluation, and definitions of PD and RD

Neck dissection specimens were categorized by cervical level using the American Joint Committee on Cancer (AJCC) cervical regional lymph node level system. Based on the histopathological results, an experienced radiologist (E.J.H.; 14 years of experience in thyroid imaging) assessed the lymph nodes on the two preoperative CT scans (obtained before the initial surgery and reoperation). All clinical and radiological data were evaluated by H.K K (11 years of experience in thyroid surgery) and E.J.H, respectively. CT data were interpreted using the Korean guidelines^3^. CT features suspicious of LN metastasis included cystic change, calcification, heterogeneous enhancement, and strong enhancement. After meticulous review of the initial CT scans and surgical and pathological records, structural disease was categorized as true RD or PD, depending on whether the lesion was identifiable on the initial scan. PD exhibited similar or identical features on the second scan, at the same location. RD was identified when the lesion was absent on the initial scan but present on the second scan.

### Response after reoperation in patients with PD or RD

An excellent response, defined as “disease-free status”, was characterized by no clinical evidence of a tumor, no evidence of a tumor by radioactive iodine (RAI) imaging and/or neck US, and a serum stimulated thyroglobulin (Tg) level < 1 ng/mL (or unstimulated Tg level < 0.2 ng/mL) in the absence of interfering antibodies^2,9^. A structurally incomplete response was defined as a tumor evident on RAI imaging and/or neck US, and cytological confirmation of malignant cells. A biochemically incomplete response was defined as a stimulated Tg level > 10 ng/mL (or unstimulated Tg level > 5.0 ng/mL) in the absence of structural disease on RAI imaging and/or neck US. An indeterminate response was defined as a stimulated Tg level of 1–10 ng/mL (or unstimulated Tg level of 0.2–5.0 ng/mL).

### Statistical analysis

Categorical variables are presented as numbers with percentages and were analyzed using the Pearson chi-squared test or Fisher’s exact test. Continuous variables are presented as means with standard deviations and were analyzed using Student’s t-test and the Mann–Whitney U test. All statistical analyses were performed using IBM SPSS Statistics for Windows (ver. 23.0.1; IBM Corp., Armonk, NY, USA). *P*-values < 0.05 were regarded as significant.
